# Continuity of GNSS as a critical attribute for safety applications in land transport

**DOI:** 10.1038/s41598-024-61937-z

**Published:** 2024-05-23

**Authors:** Aleš Filip, Francesco Rispoli

**Affiliations:** 1 University of Pardubice, Studentská 95, Pardubice, 53210 Czech Republic; 2 Radiolabs, Corso d’Italia, 19, 00198 Rome, Italy

**Keywords:** Engineering, Aerospace engineering, Electrical and electronic engineering

## Abstract

The Global Navigation Satellite System (GNSS) is widely used for air traffic management—more than 150,000 aircraft and 5000 airports worldwide are equipped with SBAS (Satellite-based augmentation system) technology, which contributes to safer and more efficient air operations. The next challenge is to extend GNSS positioning to maritime, autonomous cars and railway control systems preserving their safety requirements. The main parameter is the integrity of the GNSS positioning, although the time for which the integrity is guaranteed, defined by continuity, the most demanding requirement for aviation applications, has not been sufficiently investigated for land transportation. The aim of this paper is to close this gap by clarifying: (1) where the requirement for GNSS continuity comes from, (2) why GNSS continuity is needed in land transport, and (3) how GNSS-based applications can be made more reliable when needed. Using a comparative analysis, the continuity requirements in aviation, rail, maritime, and road transport have been investigated showing their importance for railways and automotive control, paving the way to eventually update the current EN 50126 (RAMS) and ISO/TR 4804 standards respectively for railways and automated cars. One of the main findings, through Markov modeling, is the improvement of the Mean Time to System Failure (MTTF_sys_) that for the railway safety-of-life applications can be significantly increased from about 521 h up to 5 × 10^5^ h. These results can contribute to accelerating the adoption of GNSS positioning for automated land transportation, by exploiting the extensive experience brought by the aviation sector where GNSS was introduced 20 years ago.

## Introduction

The trusted position, velocity and time (PVT) of a vehicle, preferably provided continuously, is an essential information for a safer and reliable management of incoming autonomous transportation applications. PVT information for safety applications can also be efficiently obtained using the global navigation satellite system (GNSS)^1^. It was civil aviation that first initiated the development of the GNSS safety-of-life (SoL) service in the transport sector^2^, which is mainly provided to aircraft through a regional GNSS augmentation, referred as satellite based augmentation system (SBAS)^3–5^, or a local augmentation, referred as ground based augmentation system (GBAS)^3,6^.

Over the last two decades, civil aviation has demonstrated through numerous examples that the GNSS SoL service is an efficient means to manage air safety operations, including the most demanding ones such as precision approach and landing. This has undoubtedly become a good example and motivation for the use of GNSS SoL service also in land transport—e.g. within the European railway traffic management system (ERTMS) with virtual balises detected by GNSS^7^ for safe train positioning, in maritime or river transport for navigation of vessels^8^ or more recently on roads for automated driving of cars^9^. As the GNSS SoL service was originally developed for aviation, the requirements for the GNSS signal-in-space (SIS) provided by the GNSS SoL service were specified in terms of quality attributes used in aviation.

In the 1990s, the International civil aviation organization (ICAO) defined the so-called required navigation performance (RNP) concept^10^, which includes aircraft positioning system requirements for in-flight operations (e.g. departure, en-route and approach) including the most critical operations, which are Category I, II and III precision approaches and landings^2,6^. Within the RNP, ICAO has proposed to specify the requirements for the entire navigation system using the main quality attributes: accuracy, integrity, continuity, and availability. The meaning of the above RNP attributes can be briefly described as follows. *Accuracy* is a statistical value that characterizes the positioning error in 95% of the time (2σ). *Integrity* means the ability of the system to warn the user when the system due to failures or other abnormal conditions cannot be used for safety applications. It is associated with the correctness of the provided position needed for the entire duration of the operation—which is e.g. 150 s in the case of a Category I precision approach^2^. Continuity means the probability of providing a position with the required accuracy and integrity without unscheduled interruptions during the most critical phase of the operation—which is, for example, during the 15 s before the aircraft descends to the decision height (DH of 60 m) in the case of Category I^4^. The pilot needs to decide, based on the continuous provision of (correct) PVT information during the 15 s, whether to continue the approach and landing or, e.g. due to poor visibility, to perform a backup manoeuvre (missed approach)—e.g. to fly to another airport. Availability represents the percentage of time that the system provides service within given limits—i.e., meets the requirements for accuracy, integrity and continuity.

Although GNSS meets very stringent aviation requirements^4,6^, it does not necessarily mean that it is suitable for use in other transport sectors. In this article, we will focus on GNSS continuity—its correct interpretation and use in land transport, especially in terms of meeting the requirement for reliability of PVT determination.

The aim of this article is to start from the continuity requirement set for GNSS SoL service to evaluate potential benefits of reusing this GNSS continuity attribute in other modes of transport. The goal is to increase the reliability of GNSS positioning to the level required by ground transportation. The methodology is based on (i) well-defined aeronautical RNP requirements (accuracy, integrity, continuity and availability) for the GNSS SoL service^2–5^, (ii) the interpretation of these GNSS quality metrics in terms of failure modes and associated failure probabilities^11,12^, and (iii) the use of the railway safety and dependability concept, in the sense of railway RAMS (reliability, availability, maintainability and safety)^13,14^, as a variant to the aeronautical safety concept, in the RNP sense, for comparative analysis and further investigations.

The article is arranged as follows: the second section explains the origin and meaning of continuity requirement for GNSS SoL service. Requirements for continuity of GNSS in land transport are summarised in the third section. In the fourth section, reliability analysis of GNSS-based positioning using Markov system state models is performed. The aim of the analysis is to show how the system reliability can be improved as required, even though the GNSS (subsystem) continuity itself is relatively low. The numerical results of the analysis are described in the fifth section. Finally, the practical impact of GNSS continuity analysis for land transport purposes is highlighted in the sixth section.

## Origin of continuity requirement for GNSS SoL service

The existing safety requirements for the GNSS SoL service were primarily derived from the needs of civil aviation. This is because civil aviation was the first of all modes of transport to use GNSS for traffic management. The GNSS integrity and continuity requirements, which are the main safety requirements for air navigation, were therefore directly derived from the aviation target level of safety (TLS) measured by the risk of loss of the aircraft hull over the duration of the mission^10^. The derivation of safety requirements from TLS is briefly described below.

**Figure 1:**
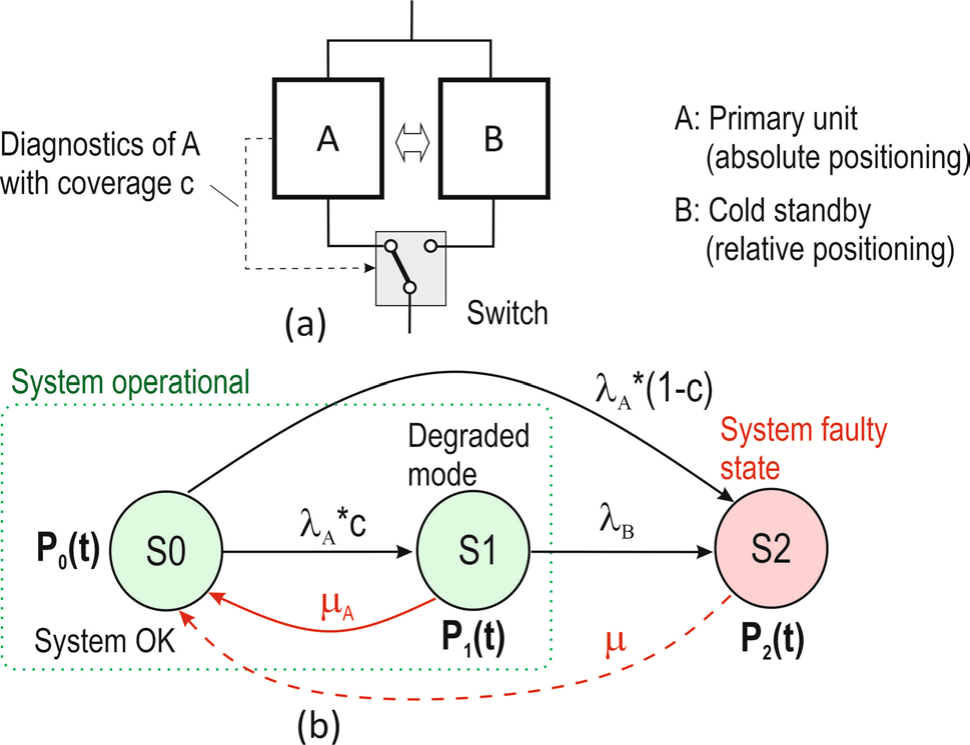
Redundant system with priority operation of unit A, cold standby B and imperfect diagnostics and switching: (**a**) schema of the system, (**b**) Markov model.

The TLS comes from ICAO historical statistical data on commercial aircraft accidents in the period 1959–1990 and was defined as a probability of hull loss (i.e. risk) of 1.5 × 10^−7^ per aircraft mission, i.e. per 1.5 h. The TLS was then allocated to each phase of the flight as well as to the final approach with a value of 1 × 10^−8^ per approach, which takes about 150 s. Since GNSS integrity and continuity are the main measures of aviation safety with respect to navigation, the TLS per approach was equally divided between integrity risk (IR), i.e. loss of integrity, and continuity risk (CR), i.e. loss of continuity, as shown in^15^ in Fig. 1.

In general, however, not every incident leads to an accident, and also in the case of loss of integrity or continuity, the pilot may be able to prevent an accident in some cases. Based on these assumptions, supplemented by specific risk reduction factors, it was derived using fault tree analysis (FTA) for non-airborne systems, i.e. GNSS SoL service, that CR is 8 × 10^−6^ per 15 s and IR is 2 × 10^−7^ per approach (i.e. 150 s)^15^. CR is equal to the continuity (C) complement to 1, i.e. C = 1 $-$ CR, and IR is equal to the integrity (I) complement to 1, i.e. I = 1 $-$ IR. The same FTA shows that the CR requirement for airborne equipment, i.e. GNSS receiver and accessories, is 2 × 10^−6^ per 15 s.

Continuity is the ability of the system or service to provide navigation accuracy and integrity (integrity is monitored) throughout the intended operation given that the navigation accuracy and the integrity are provided at the start of the operation^16–19^. Continuity is a quality measure whether the system is functioning when it is really needed. This corresponds to the reliability (i.e. probability of correct functioning) of the system, which is related to a specific operation of usually short duration—e.g. 15 s or 1 h in aviation. Therefore, e.g. the continuity requirements for the provision of GNSS signal-in-space (SIS) for typical air operations can be considered as a (short-term) reliability of service^19^. For comparison, the railway standard EN 50126-1^13^ defines reliability as follows: ‘ability to perform as required, without failure, for a given time interval, under given conditions’. So how the aviation continuity differs from reliability on railways? Railway reliability does not refer to the duration of a typical operation because on rail, unlike aviation, the duration of an operation would be difficult to determine.

The reliability of an item (system) may be measured in different ways, depending on the situation, e.g. as: mean time to failure (MTTF) for non-repairable items, mean time between failures (MTBF) for repairable items, failure rate (λ) or the probability of correct item functioning R(t) = exp(−λ t) as a function of time *t*. The mean time to restore (MTTR) is usually much smaller than the lifetime of the item, then the values of MTTF and MTBF are practically the same, because MTBF = MTTF + MTTR. With this simplification, one can also write for constant failure rate that λ  = (MTTF)^−1^ = (MTBF)^−1^. The assumption of a constant failure rate is often used in reliability analyses of technical systems.

Continuity means the reliable operation of the system during a certain continuity time interval (CTI). As GNSS is a repairable system, then the SoL service continuity can be expressed as Refs.^16,18^

1 $$C = {\text{exp}}\left( { - \frac{CTI}{{MTBF}}} \right)$$

This is the standard expression for reliability and excludes scheduled outages (i.e. uses random parameter MTBF) assuming that planned outages will be notified and the operation will not take place. If ${\text{CTI}}\ll {\text{MTBF}}$, then

2 $$C \cong 1 - CTI/MTBF$$

Continuity risk (CR) is the probability that the GNSS system will be unintentionally interrupted and will not provide navigation outputs with the required quality over the intended period of time (CTI), assuming that the outputs were present with specified quality at the beginning of a given operation. This occurs when the integrity monitor of SBAS or GBAS triggers a true alert, a false alert, or does not have enough information to make a decision. CR here refers to unplanned interruptions of GNSS service. Loss of GNSS signal-in-space (SIS) due to obstructions along a railway line or road is not a loss of continuity because correct functioning of GNSS positioning can be well predicted from the profile of the surrounding environment. Since CR equals the continuity complement to 1, the expression (2) yields

3 $$CR = CTI/MTBF$$

The aviation GNSS SoL service CR requirement of 8 × 10^−6^ per 15 s for a Category I precision approach can be converted to an MTBF of 520.83 h using (3). For the sake of completeness, this continuity requirement was not introduced only with the coming of GPS technology, but before that for the so-called instrument landing system (ILS)^6,20^. Current GNSS receivers integrated with GNSS antennas achieve MTBF > 50000 h, therefore, in further considerations below in “Reliability analysis of GNSS-based positioning”, we neglect the GNSS receiver reliability with respect to much worse SoL service reliability. The primary focus on assessing the impact of GNSS SoL reliability can also be justified by the fact that the quality of service affects many GNSS users, whereas the quality of the GNSS receiver affects only one user. Although continuity is always related to the accuracy and integrity of positioning, this article focuses on GNSS SoL service continuity and its impact on the reliability of positioning in land transport.

## Continuity requirements for GNSS in land transport

In Europe, the strategy to adopt GNSS for the transport sector is based on the use of EGNSS: the Galileo satellite navigation system and the EGNOS SoL service developed for aviation. In that scenario, in addition to the integrity of accuracy, the key to the efficient operational use of the EGNOS SoL service becomes the continuity of the service provided. For what purpose would be an early warning to the user that the GNSS SoL service is not able to provide the required accuracy of the positioning function (and thus ensure integrity) if unplanned service outages do not allow its intended use—e.g. virtual balise detection in the case of ERTMS or continuous positioning of a self-driving car in the overtaking phase. Maritime transport is the only surface transport sector that has historically set requirements for continuity of GNSS SoL service^21,22^. It is also the sector that has a safety concept most similar to that of air transport—as maritime safety is directly dependent on both safety integrity and reliability (and availability). This section therefore first analyses the importance of GNSS SoL service continuity for maritime navigation purposes. This is followed by an analysis of the applicability of continuity from a railway perspective. The railway has very well-defined safety and dependability requirements (RAMS) for safety-related systems. Yet, the requirement for GNSS SoL continuity is not explicitly required on the railway^13,14,23^. Finally, a brief mention is made of the possible need for GNSS SoL service continuity in the area of self-driving cars, which is currently the most dynamically developing area of transport.

### Continuity requirements for maritime

User requirements for GNSS-based maritime navigation were specified in IMO Resolution A.915(22)^21^ two decades ago. Most of the requirements for the categories of navigation in oceans, coastal waters, harbour approaches and restricted waters specify an accuracy of 10 m (95%). Accuracy of 1 m or less is required for port operations—e.g. 0.1 m accuracy for automatic docking. Practically the same approach for the specification of GNSS-based navigation requirements as is applied in aviation has been implicitly used in the maritime sector. This means that maritime GNSS requirements are defined for each category of operation in terms of accuracy, integrity, continuity, and availability.

Integrity and continuity are defined in the maritime sector over the duration of an operation, i.e. a specific manoeuvre such as entering a port or docking. It should be reminded here that in aviation, integrity is defined for the duration of the entire operation, e.g. 150 s for a precision Category I approach, and continuity is defined for the most critical phase of that operation, e.g. 15 s before reaching the decision height. For some flight operations, e.g. en-route or non-precision approach (NPA), both integrity and continuity are defined on a one-hour basis because it is not possible to simply estimate the average duration of flight operations. In maritime the problem is that, unlike aviation, IMO A.915(22)^21^ completely lacks a rationale for how the maritime requirements for GNSS were derived. A broadly acceptable maritime safety goal for this derivation is missing. It is not explained in how the risk of integrity (IR) and continuity (CR) in terms of the failure probability and the associated duration of operation were derived, i.e. IR of 1 × 10^−5^ per 3 h and CR of 3 × 10^−4^ per 3 h (i.e. continuity of 99.97% per 3 h according to the original IMO format^21^). In terms of correctness, however, it should be noted that an attempt was subsequently made to retrospectively justify the derivation of the IR and CR attributes using the TLS defined for maritime operations^11^, but this attempt has not been adopted by the maritime community.

Over time it became apparent that the initial maritime requirement for continuity (1–3 × 10^−4^ per 3 h) was very strict due to very long continuity time interval (CTI) and not achievable by GNSS technology. Translating the maritime continuity requirement in terms of a CR of 3 × 10^−4^ per 3 h for the CTI of 15 s used in aviation gives a CR of 4.2 × 10^−7^ per 15 s. For comparison, e.g. the actual EGNOS SoL service performance in terms of continuity for localizer performance with vertical guidance (LPV-200/ CAT I) and approach with vertical guidance I (APV-I) flight operations is better than 1–1 × 10^−4^ per 15 s in the core of ECAC (European Civil Aviation Conference) region^4^. It is therefore clear that the initial maritime requirement for GNSS continuity is unrealistic. For this reason, the CTI was implicitly reduced from 3 h to 15 min—as stated in IMO resolution A.1046(27)^22^. The updated maritime CR requirement of 3 × 10^−4^ per 15 min and translation to a 1 h basis gives a CR of 1.2 × 10^−3^ per 1 h. This according to (3) equals to an MTBF of 833.33 h. If a CTI of 15 s is used, then the translation of this new maritime requirement for continuity is 1–5 × 10^−6^ per 15 s. This corresponds roughly to the aeronautical requirement for GNSS continuity, i.e. 1–8 × 10^−6^ per 15 s. The new multi-constellation and multi-frequency EGNOS V3 (2025) is expected to meet aviation continuity requirements and therefore the new maritime GNSS continuity requirement can be considered realistic.

### Reliability requirements for rail

The basic framework for ensuring the safety and dependability of railway systems is defined in the CENELEC standards EN 50126-1^13^ and EN 50126-2^14^ on the specification and demonstration of RAMS. These standards consider the railway system in a given physical and operational environment, i.e., including human operators, as well as the factors that influence the railway RAMS—in particular the technical system and the operating and maintenance conditions. The framework defined by the RAMS can be imagined as an umbrella under which a safety-related system is subsequently developed and implemented according to the downstream standards EN 50129^24^ (safety-related system), EN 50128^25^ (software for safety-related system), and others. The safety of the railway signalling system is based on three main pillars: (1) functional safety—i.e. mainly safety integrity (S) of each safety function designed to mitigate a specific hazard, (2) technical safety—i.e. a prescribed safe behaviour of the system in case of a dangerous failure, and (3) high dependability—i.e. reliability, availability and maintainability (RAM), because occasional irregularities in train operations due to degraded operational mode of signalling system with participation of a human factor may indirectly compromise railway safety.

As the safety concepts in aviation and on railways are different, in general the aviation requirements for GNSS SoL services cannot be directly applied to the design and approval of safety-related systems on railways. The opposite approach should be taken, i.e. first define the requirements for a safe and reliable train positioning function based on the GNSS SoL service for the application in terms of the railway RAMS and safety standards EN 5012 × and other regulations, and then use the relevant GNSS service (e.g. EGNOS and/or GBAS) and other sensors and techniques to meet these railway requirements. The key to success is the correct interpretation and assurance of the aeronautical RNP attributes for GNSS in terms of railway RAMS^7,12^.

As evident from above, continuity as a quality measure of safety systems or service is not contained in the railway EN 50126 RAMS framework as a safety parameter. Nevertheless, continuity is an important quality attribute of GNSS SoL service performance, which also significantly affects cost of GNSS SoL service, and therefore continuity cannot simply be omitted when designing GNSS-based railway systems.

EN 50126^13,14^ prescribe a well-developed method to specify system requirements based on a risk assessment process. The following principles for risk acceptance can be used: codes of practice (CoP), similar reference systems or explicit risk estimation. If there is sufficient experience with a given railway safety-related system, which is also the case of ERTMS, then CoP (i.e. CENELEC standards, ERTMS technical specifications for interoperability (TSIs), EU and national regulations and other documents) can be used to specify the requirements for ERTMS based on GNSS. The ERTMS TSIs contain, among others, the RAMS requirements for the on-board and trackside part, including requirements for the track balise and onboard balise transmission module (BTM) used for safe train position determination. ERTMS TSIs have been used to specify the safety requirements for GNSS-based virtual balise detection^23,26^. Similarly, ERTMS specification^27^ can be used to determine the reliability requirements for virtual balise detection.

The ERTMS mission reliability targets^27^ consist of qualitative and quantitative requirements. The quantitative requirements are expressed in MTBF and are differentiated according to the criticality (immobilising, service or minor) of the considered failures, as shown in Table 1. In the ERTMS context, immobilising failures are identified as all the ERTMS failures, which cause two or more trains to be switched in on-sight mode (i.e. driver’s responsibility). Service failures cause the nominal performance of one or more trains to be reduced and/or at most one train to be switched in on-sight mode. A minor hardware (HW) failure is a failure which results in excessive unscheduled maintenance and cannot be classified as immobilising or service failure.

**Table 1:** Reliability requirements for the European railway traffic management system^27^.

	Reliability of ERTMS in MTBF [hours]		
Type of failure	On-board equipment	Central track-side equipment	Line-side equipment
Immobilizing failures	2.7 × 10^6^	3.5 × 10^8^	1.2 × 10^5^
Service failures	3.0 × 10^5^	4.0 × 10^7^	1.4 × 10^4^
Minor HW failures	8.0 × 10^3^	1.0 × 10^5^	3.6 × 10^2^

It is stated in the ERTMS/ETCS Subset 36^28^ that the minimum operational lifetime of a track balise should be 20 years. ERTMS tenders often contain MTBF values of 50,000 + h for a complete ERTMS on-board subsystem and MTBF of 50 + years for ERTMS balises^23^. If a virtual balise detected by GNSS should replace a fixed track balise, then the MTBF of the virtual balise detection should be about 5 × 10^5^ h (1 year $\cong$ 10^4^ h). MTBF of 5 × 10^5^ h required for an ERTMS balise exceeds the MTBF of 520.83 h required by aviation from the GNSS SoL service by approximately 3 orders of magnitude. Both of these MTBF values are used as major inputs in the reliability analysis in section below.

### GNSS continuity for automated car driving

In the field of automated driving systems (ADS) for cars there is currently still no consensus on the need to use the GNSS continuity. Therefore, only two opposing views on the applicability of continuity for ADS are presented below.

Let’s first take a closer look at the problem in terms of the relevant automotive safety standards. A safety function mitigating risk can be considered safe if ISO 26262 (Automotive functional safety)^29^ and ISO/PAS 21448 (safety of the intended functionality—SOTIF)^30^ standards are used for its design and implementation. However, vehicles cannot be in a safe state without secure operation specified in the standard ISO SAE 21434. To cover the whole area of ADS safety, standard ISO/TR 4804 (Road vehicles—Safety and cybersecurity for automated driving systems—Design, verification and validation)^31^ was recently developed. The intention of ISO/TR 4804 is to put together standards ISO 26262, ISO/PAS 21448 and ISO SAE 21434 under one risk-based approach. ISO/TR 4808, which is the umbrella for all other automotive safety standards, states that the continuity metric is no longer the main parameter of GNSS-based positioning with integrity. This is justified by the fact that GNSS based positioning cannot have high continuity due to environmental obstructions of GNSS signal-in-space, such as bridges or tunnels. However, this claim is in conflict with the definition of continuity, which is measured by unscheduled positioning outages. Loss of GNSS signal-in-space due to obstructions around a railway line or road can be well predicted and is therefore not related to loss of continuity of service.

Completely different views on the need for GNSS continuity for safety-critical applications in the automotive and other transport sectors are given in the GNSS user technology report^1^, where GNSS continuity is considered a high priority requirement. The above-mentioned views on the use of GNSS continuity in automotive transport are quite different and therefore need to be monitored further.

## Reliability analysis of GNSS-based positioning

The aim of the analysis is to show how to meet the strict railway (ERTMS) requirement for reliability of GNSS-based train positioning in terms of MTBF of 5 × 10^5^ h (as it was specified above) although the reliability (continuity) of the GNSS SoL service is relatively very low. At the same time, this analysis can be seen as a guide to improve the reliability of GNSS-based positioning of transport means in other land transport applications. In maritime, e.g., resilient positioning solutions based on GNSS and other diverse sensors have been investigated for continuity and integrity using fault tree analysis (FTA)^32^. The disadvantage of the FTA method is that it does not include time analysis of system faulty states, which is required for rail applications. Therefore, for the reliability analysis in this paper, Markov analysis was used^33^, which, unlike FTA, allows to efficiently solve the time dependencies of the probabilities of fault or fault-free states of the system, including the calculation of the MTTF.

**Figure 2:**
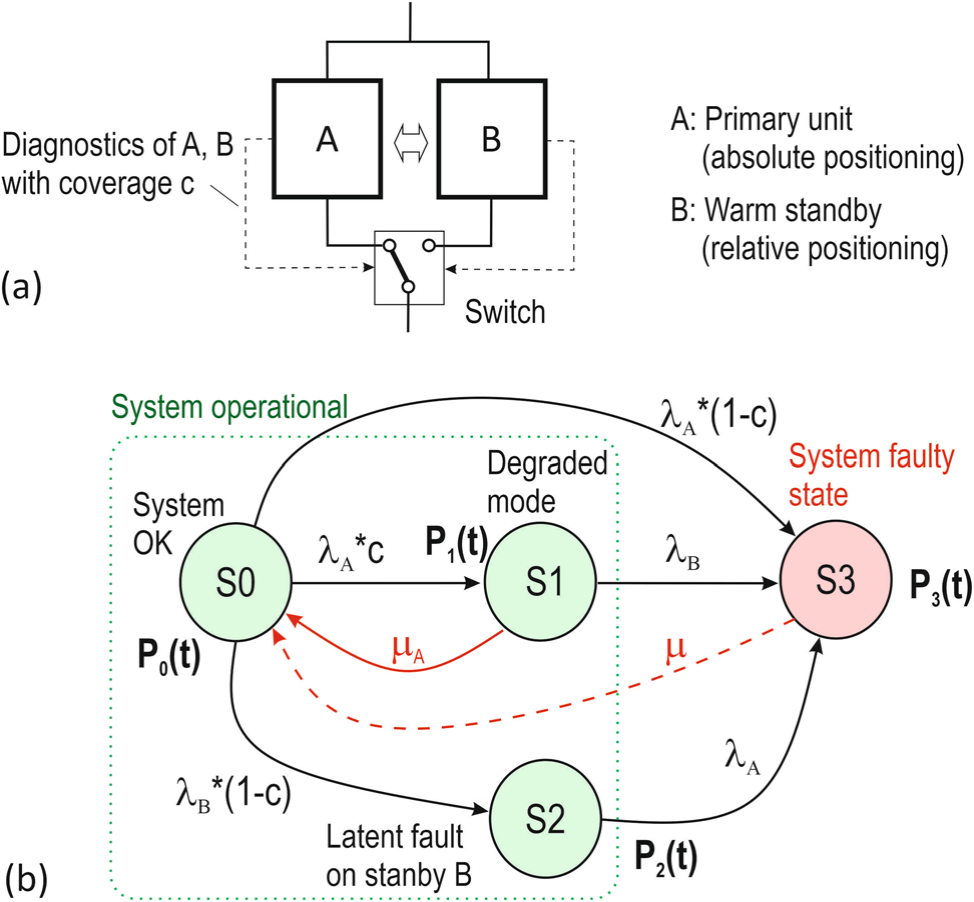
Redundant system with priority operation of unit A, warm standby B and imperfect diagnostics and switching: (**a**) schema of the system, (**b**) Markov state model.

For this purpose, redundant 1oo2 (one-out-of-two) architectures with primary unit A and standby B are used—as shown in Figs. 1a and 2a. Unit A is based on GNSS and provides an absolute position. Backup B is an inertial measurement unit (IMU) that provides a relative position. Fault diagnostics is critical, as it ensures the correct switchover of operation from the failed primary unit A to the backup B. When the correct function of unit A is restored, the operation is switched from standby B to unit A.

For simplicity, assume that the EGNOS V3 service meets the aeronautical continuity requirement for a Category I precision approach in terms of a continuity risk (CR) of 8 × 10^−6^ in 15 s, which according to (3) corresponds to an MTBF of 520.83 h and a CR (i.e. also failure rate) of 1.92 × 10^−3^ in 1 h. Therefore, let the MTBF of unit A (MTBF_A_) be 520.83 h. The MTBF of the B unit (MTBF_B_) will be variable to meet the reliability requirement for the ERTMS virtual balise detection (MTBF of 5 × 10^5^ h).

The reliability analysis is demonstrated using two examples of redundant architectures and the corresponding Markov models in Figs. 1b and 2b. Markov models describe the system through system states, e.g. S0, S1, S2 etc., with transitions between them depending on failure rates (λ) and restore/ repair rates (μ). The states must be mutually exclusive and collectively exhaustive. Based on the state model, the time dependencies of the probabilities of each state, e.g. P_0_(t), P_1_(t), P_2_(t) etc., in which the system is found are calculated. Using these probabilities, reliability, availability, failure rate, and other system attributes can be determined.

The goal in this paper is to calculate the mean time to failure (MTTF_sys_) for the system architecture using Markov models^33^. Although a GNSS-based positioning system for transport applications is considered to be repairable, the resulting calculated reliability is reported in this paper in terms of MTTF_sys_ and not MTBF_sys_. This is because, as shown below in “ Example 1”, the output attribute when analysing the reliability of systems using Markov models is the MTTF. However, as shown above, the values of MTTF and MTBF are almost identical. For the architectures in Figs. 1 and 2, the function of the primary unit A is assumed to be superior to that of unit B. These are therefore Markov models with priority of operation. The examples are given below.

### Example 1: Redundant system with priority operation of unit A, cold standby B, imperfect diagnostics of unit A, and online restoration of unit A

The architecture of the basic redundant system with priority operation of one of the units is shown in Fig. 1a. The primary unit A is GNSS-based and provides absolute positioning. The standby B provides a relative position. Units A is assumed to have imperfect diagnostics with diagnostic coverage c (probability of fault detection). Diagnostics of the standby B is performed only off-line in this example.

For the Markov model of the architecture in Fig. 1b, the following three system states are defined:

- S0: Fully functional system state. Primary unit A is operating according to specifications. Standby unit B is fault-free and not operational.
- S1: Degraded operational mode. It is still functional state. Primary unit A is faulty. Fault on unit A is detected by imperfect online diagnostics and is restored with a frequency of μ_A_. Switchover to standby unit B is successful. Standby B is operational.
- S2: System faulty state. It is the result of a fault on standby B or when the diagnostics of faulty unit A fails. Recovery of the system with a frequency μ will bring the system from state S2 to S0.

The corresponding time-dependent state probabilities according to the Markov model in Fig. 1b are P_0_(t), P_1_(t) and P_2_(t)$.$ The intention is to determine the MTTF_sys_. It can be calculated by integrating the system reliability R(t) = P_0_(t) + P_1_(t) over time t from zero to infinity for non-absorbing states S0 and S1. A prerequisite for a correct system reliability calculation is that the faulty state of the system S2 must be an absorbing state. The absorbing faulty state means that, once entered, cannot be left until the system is correctly restored. Therefore, in the case of MTTF_sys_ calculation according to Fig. 1b, the (dashed) directional arc indicating the system restoration with frequency μ must be omitted. Mean time to restore/ repair (MTTR), which is indirectly proportional to μ(μ = 1/MTTR), can be used to calculate system availability as A = MTTF_sys_/(MTTF_sys_ + MTTR). Thus, MTTF_sys_ characterizes, besides reliability, also the availability of the system. Online restoration of primary unit A with frequency μ_A_ can bring the system from degraded state S1 to the fully operational state S0. Therefore, online diagnostics and restoration is the key to increase system reliability and MTTF. The Markov model for the system according to Fig. 1b can be described by the system of linear differential equations as

4 $$\left( {\begin{array}{*{20}c} {\dot{P}_{0} \left( t \right)} \\ {\dot{P}_{1} \left( t \right)} \\ {\dot{P}_{2} \left( t \right)} \\ \end{array} } \right) = \left( {\begin{array}{*{20}c} { - [\lambda_{A} c + \lambda_{A} \left( {1 - c} \right)]} & {{\mu}_{A} } & 0 \\ { \lambda_{A} c} & { - \left( {\mu_{A} + \lambda_{B} } \right)} & 0 \\ { \lambda_{A} \left( {1 - c} \right)]} & {\lambda_{B} } & 0 \\ \end{array} } \right) \cdot \left( {\begin{array}{*{20}c} {P_{0} \left( t \right)} \\ {P_{1} \left( t \right)} \\ {P_{2} \left( t \right)} \\ \end{array} } \right),$$

with the initial conditions for the state probabilities [P_0_(0), P_1_(0), P_2_(0)] = (1,0,0). To calculate the mean time to system failure (MTTF_sys_), we use the Laplace transform (marked as *) and the limit theorem as follows

5 $$MTTF_{j} = \mathop \smallint \limits_{0}^{\infty } P_{j} \left( t \right)dt = \mathop {{\text{lim}}}\limits_{s \to 0} \mathop \smallint \limits_{0}^{\infty } P_{j} \left( t \right)e^{ - st} dt = P_{j}^{*} \left( 0 \right) MTTF_{sys} = \sum P_{j}^{*} \left( 0 \right) ,$$

where j represents the non-absorbing states. The system of Eqs. (4) after the Laplace transform and application of the initial conditions is﻿

6 $$\left( {\begin{array}{*{20}c} {sP_{0}^{*} \left( s \right) - 1} \\ {sP_{1}^{*} \left( s \right) - 0} \\ {sP_{2}^{*} \left( s \right) - 0} \\ \end{array} } \right) = \left( {\begin{array}{*{20}c} { - [\lambda_{A} c + \lambda_{A} \left( {1 - c} \right)]} & {\mu_{A} } & 0 \\ { \lambda_{A} c} & { - \left( {\mu_{A} + \lambda_{B} } \right)} & 0 \\ { \lambda_{A} \left( {1 - c} \right)]} & {\lambda_{B} } & 0 \\ \end{array} } \right) \cdot \left( {\begin{array}{*{20}c} {P_{0}^{*} \left( s \right)} \\ {P_{1}^{*} \left( s \right)} \\ {P_{2}^{*} \left( s \right)} \\ \end{array} } \right),$$

Since the complex frequency domain parameter s → 0, then $s{P}_{j}^{*}\left(s\right)$→ 0. For the MTTF_sys_ calculations, only the equations for the non-absorbing states (j = 0, 1) are used from (6) as follows

7 $$\left( {\begin{array}{*{20}c} { - 1} \\ { 0} \\ \end{array} } \right) = \left( {\begin{array}{*{20}c} { - [\lambda_{A} c + \lambda_{A} \left( {1 - c} \right)]} & {\mu_{A} } \\ {\lambda_{A} c} & { - \left( {\mu_{A} + \lambda_{B} } \right)} \\ \end{array} } \right) \cdot \left( {\begin{array}{*{20}c} {P_{0}^{*} \left( 0 \right)} \\ {P_{1}^{*} \left( 0 \right)} \\ \end{array} } \right),$$

Solving the system of Eqs. (7) gives

8 $$P_{0}^{*} \left( 0 \right) = \frac{{\mu_{A} + \lambda_{B} }}{{\lambda_{A} \cdot [\lambda_{B} +\mu_{A} \left( {1 - c} \right)]}},$$

9 $$P_{1}^{*} \left( 0 \right) = \frac{c}{{\lambda_{B} +\mu_{A} \left( {1 - c} \right)}},$$

and using (5) the mean time to (first) system failure is

10 $$MTTF_{sys} = P_{0}^{*} \left( 0 \right) + P_{1}^{*} \left( 0 \right) = \frac{{\mu_{A} + \lambda_{B} + \lambda_{A} \cdot c}}{{\lambda_{A} \cdot [\lambda_{B} +\mu_{A} \left( {1 - c} \right)]}}$$

Note: The diagnostic coverage c in (10) refers only to the primary unit A. In “Results and discussion” , for clarity, this coverage is denoted as c(A).

### Example 2: Redundant system with priority operation of unit A, warm standby B, imperfect diagnostics of units A and B, online restoration of unit A

The system architecture in this example is shown in Fig. 2a and the corresponding Markov model is shown in Fig. 2b. The difference of this architecture with respect to the architecture in Fig. 1 is that both units (A and B) are equipped with online diagnostics. Since the diagnostics of faulty standby B with coverage c can fail, the Markov model additionally contains state S2—latent fault of standby B.

For the Markov model in Fig. 2b, the following four states of the system can be defined:

- S0: Fully functional system state. The GNSS-based primary unit A is operating according to specifications. Fault-free standby B (e.g. IMU) is not operational.
- S1: Degraded mode of operation, but functional system state. Fault of unit A is detected by diagnostics with coverage c and is recovered online with a frequency of μ_A_. The switchover to standby B was successful. Standby unit B is operational.
- S2: Standby B has a latent fault.
- S3: System faulty state. This occurs when: (i) a fault of primary unit A is not detected, (ii) unit A fails and then standby B fails, or (iii) standby B has a hidden fault and then unit A fails.

The Markov model in Fig. 2b can be described by the system of linear differential equations as

11 $$\left( {\begin{array}{*{20}c} {\dot{P}_{0} \left( t \right)} \\ {\dot{P}_{1} \left( t \right)} \\ {\dot{P}_{2} \left( t \right)} \\ {\dot{P}_{3} \left( t \right)} \\ \end{array} } \right) = \left( {\begin{array}{*{20}c} { - [\lambda_{A} c + \lambda_{A} \left( {1 - c} \right) + \lambda_{B} \left( {1 - c} \right)]} & {\mu_{A} } & 0 & 0 \\ { \lambda_{A} c} & { - \left( {\mu_{A} + \lambda_{B} } \right)} & 0 & 0 \\ { \lambda_{B} \left( {1 - c} \right)} & 0 & { - \lambda_{A} } & 0 \\ { \lambda_{A} \left( {1 - c} \right)} & { \lambda_{B} } & { \lambda_{A} } & 0 \\ \end{array} } \right) \cdot \left( {\begin{array}{*{20}c} {P_{0} \left( t \right)} \\ {P_{1} \left( t \right)} \\ {P_{2} \left( t \right)} \\ {P_{3} \left( t \right)} \\ \end{array} } \right),$$

with the initial conditions for the state probabilities [P_0_(0), P_1_(0), P_2_(0), P_3_(0)] = (1,0,0,0). Solving the mean time to system failure using (11) and the Laplace transform described in Example 1 gives

12 $$MTTF_{sys} = \frac{{\left( {\mu_{A} + \lambda_{B} } \right) \cdot \frac{{\left[ {\lambda_{A} + \lambda_{B} \left( {1 - c} \right)} \right]}}{{\lambda_{A}^{2} \cdot c}} + 1}}{{\{ [\lambda_{A} + \lambda_{B} \left( {1 - c} \right)] \cdot \frac{{\mu_{A} + \lambda_{B} }}{{\lambda_{A} \cdot c}} -\mu_{A} \} }}$$

Note: The diagnostic coverage c in (12) refers to units A and B. In “Results and discussion” this coverage is denoted as c(A, B) for clarity.

## Results and discussion

This section describes the reliability calculations and discusses the results achieved in terms of MTTF_sys_ for systems based on the aviation GNSS SoL service and intended for vehicle positioning in land transport. The calculations are performed according to the expressions derived for redundant architectures in the previous section. In particular, the aim of these calculations is to show how the relatively low reliability (continuity) of the GNSS SoL service-based positioning can be increased to the level of reliability acceptable in rail transport. The railway case is considered because the reliability requirements for positioning of trains are the highest of all the surface transport modes mentioned.

The following symbols are used in the tables below: CR_A_—is the required aviation continuity risk or real performance for GNSS SoL service considered in channel A of the redundant architecture, MTBF_A_—is the mean time between failures of unit A corresponding to the continuity risk CR_A_, λ_A_—is the failure rate per 1 h of unit A corresponding to the continuity risk CR_A_ (λ_A_ = 1/MTBF_A_), MTBF_B_—is the selected mean time between failures of unit B, μ_A_—is the recovery frequency of unit A per 1 h, λ_B_—is the failure rate per 1 h of unit B corresponding to the MTBF_B_, c(A)—is the diagnostic coverage (probability of failure detection) of unit A, and c(A, B)—is the diagnostic coverage of units A and B. For the sake of simplicity in this section, we assume that the MTBF and MTTF values are practically identical for relatively short MTTR, because MTBF = MTTF + MTTR. The input reliability values of units A and B are considered in terms of MTBF and associated failure rates per 1 h.

Table 2 contains the calculated MTTF_sys_ values according to (10) for the architecture in Fig. 1. The main objective here is to show the effect of diagnostic coverage c(A) of the primary unit A on the overall system reliability. Unit A represents the GNSS-based positioning and unit B represents the IMU-based backup relative positioning. Thus, the operation of unit A is prioritized over the operation of standby B. In the calculation of MTTF_sys_, only the continuity of the GNSS SoL service is considered, which is measured by the continuity risk CR_A_ in terms of (3). Since continuity is one of the most demanding quality attributes of GNSS SoL service to achieve, in Table 2 we consider both the aviation continuity risk requirement for safety operations, e.g., APV I/ LPV-200/ CAT I—i.e., 8 × 10^−6^ per 15 s, and the actual EGNOS SoL service performance in terms of achieved continuity risk—i.e., 1 × 10^−4^ per 15 s in the core of ECAC region^4^. The CR_A_ value is converted to MTBF_A_ using (3) and also to λ_A_ using the expression λ_A_ = 1/MTBF_A_. The CR_A_ of 1 × 10^−4^ per 15 s corresponds to the MTBF_A_ of 41.66 h. For standby B, a MTBF_B_ of 1000 h was first selected, which is approximately twice the MTBF value for the GNSS SoL service. The average duration of a train mission is 1 h^34^, and therefore the corresponding minimum recovery frequency of unit A (μ_A_) is 1 recovery per 1 h. The calculated MTTF_sys_ values strongly depends on the diagnostic coverage c(A) of unit A. Table 2 shows that the system in Example 1 is unable to meet the railway MTBF requirement of 5 × 10^5^ h for a real CR performance of EGNOS. The railway MTBF requirement can be met, e.g., for the following input values: a CR_A_ of 8 × 10^−6^ per 15 s, an MTBF_B_ of 1000 h, μ_A_ of 1 restoration per 1 h, and a high diagnostic coverage c(A) of 0.99999.

**Table 2:** Effect of diagnostic coverage c(A) of unit A on MTTF_sys_ according to Example 1.

CR_A_ [per 15 s]	MTBF_A_ [h]	λ_A_ [per h]	MTBF_B_ [h]	λ_B_ [per h]	μ_A_ [per h]	c (A) [−]	MTTF_sys_ (10) [h]
8 × 10^−6^ aviation requirement	520.83	1.92 × 10^−3^	1000	1 × 10^−3^	1	1	5.22 × 10^5^
						0.99999	5.17 × 10^5^
						0.9999	4.75 × 10^5^
						0.999	2.61 × 10^5^
						0.99	4.75 × 10^4^
1 × 10^−4^ EGNOS performance	41.66	2.40 × 10^−2^	1000	1 × 10^−3^	1	1	4.27 × 10^4^
						0.99999	4.23 × 10^4^
						0.9999	3.88 × 10^4^
						0.999	2.14 × 10^4^
						0.99	3.88 × 10^3^

Table 3 contains the MTTF_sys_ values calculated using (12) for the architecture with priority operation of unit A with warm standby B shown in Fig. 2. The main objective is to show to what extent the system reliability is affected when both units of the system are equipped with online diagnostics with diagnostic coverage c(A, B), which is shown in Table 3, compared to a system where only unit A is equipped with online diagnostics with coverage c(A), which is shown in Table 2. For example, from comparing the calculated MTTF_sys_ value of 2.07 × 10^5^ h in Table 3 and the MTTF_sys_ value of 2.61 × 10^5^ in Table 2 for the identical input values, including diagnostic coverage (0.999), the imperfect diagnostics with coverage c(A, B) in Example 2 slightly reduces the MTTF_sys_ value relative to the applied diagnostics with coverage c(A) in Example 1. This is understandable because perfect diagnostics is replaced with imperfect diagnostics. A further increase in MTTF_sys_ can be achieved by increasing the input values of MTBF_B_ and μ_A_, as can be seen in Table 3.

**Table 3:** Effect of diagnostic coverage c(A, B) of primary unit A and standby unit B on MTTF_sys_ according to Example 2.

CR_A_ [per 15 s]	MTBF_A_ [h]	λ_A_ [per h]	MTBF_B_ [h]	λ_B_ [per h]	μ_A_ [per h]	c (A, B) [−]	MTTF_sys_ (12) [h]
8 × 10^−6^	520.83	1.92 × 10^−3^	1000	1 × 10^−3^	1	0.9999	4.53 × 10^5^
						0.999	2.07 × 10^5^
						0.99	3.24 × 10^4^
			1000	1 × 10^−3^	10	0.9999	2.07 × 10^6^
						0.999	3.22 × 10^5^
						0.99	3.42 × 10^4^
			10000	1 × 10^−4^	1	0.9999	2.54 × 10^6^
						0.999	4.53 × 10^5^
						0.99	4.92 × 10^4^
			10000	1 × 10^−4^	10	0.9999	4.52 × 10^6^
						0.999	4.90 × 10^5^
						0.99	4.95 × 10^4^
			20000	5 × 10^−5^	1	0.9999	3.42 × 10^6^
						0.999	4.85 × 10^5^
						0.99	5.06 × 10^4^
			20000	5 × 10^−5^	10	0.9999	4.84 × 10^6^
						0.999	5.05 × 10^5^
						0.99	5.08 × 10^4^

Based on the calculated MTTF_sys_ values shown in Tables 2 and 3, it can be concluded that the reliability of the position determination function based on the aviation GNSS SoL service can be significantly improved by using a standby unit such as an IMU. In this case, it is a redundant system with priority operation of unit A providing absolute positioning and standby unit B providing relative positioning. In order to achieve the required MTTF_sys_, e.g. 5 × 10^5^ h, which is the railway reliability requirement for ERTMS, the system based on GNSS SoL service must be equipped with a reliable standby unit B (e.g. MTBF_B_ of 10000 h or more) and high quality online diagnostics and unit switching.

## Impact

The potential benefits of re-using the continuity performance guaranteed by the EGNOS SoL service are a better reliability of the position determination function especially for use cases where the position of the vehicle must be determined continuously during the undertaken operation. This is the case of autonomous train operation (ATO) for rail and the lane keeping function for autonomous vehicles. The increase of reliability is important for both classes of applications. In addition to the aviation and maritime sectors, continuity is also a critical quality attribute of GNSS SoL service for safe and dependable land transport.

## Conclusions

The railway safety and dependability concept based on the standard EN 50126 (RAMS) does not directly specify continuity requirements for GNSS, but there are very demanding requirements for system reliability, e.g. for ERTMS, as reliability indirectly affects railway safety. European railways aim to use the GNSS SoL service, in particular EGNOS, which was developed for aviation, and to benefit as much as possible from its high quality in the sense of a railway RAMS. Further, it appears that there is no consensus in the automotive industry on the use of GNSS continuity. For example, the current automotive standard ISO/TR 4804 does not recommend using GNSS continuity as the main quality attribute for GNSS-based positioning with integrity. We show that this recommendation is based on a misunderstanding of the GNSS continuity concept. As the railway has the most stringent requirements for system reliability in surface transport, the MTBF value of 5 × 10^5^ h, which is required for the detection of the ERTMS virtual balise by GNSS, was chosen as the reliability target for the analysis described in “Reliability analysis of GNSS-based positioning”.

According to reliability theory, the MTTF of systems can be increased by using redundant architectures. Therefore, in this paper, simplified 1oo2 (one-out-of-two) architectures have been analysed, where channel A is represented by the GNSS SoL service with guaranteed continuity for aviation (corresponding to an MTBF of 520.83 h) and channel B is implemented by e.g. IMU. The reliability of the GNSS receiver and antenna, which is several times greater than the reliability of the SoL service, has been neglected for simplicity in this analysis. Systems of differential linear equations based on Markov models of the corresponding architectures, the Laplace transform, and the limit theorem were used to numerically solve the mean time to system failure (MTTF_sys_). The numerical results of MTTF_sys_ presented in “Results and discussion” demonstrate that redundant architectures when using aviation GNSS SoL service will enable to meet the high reliability requirements of railways for safe GNSS-based train localization.

## Data Availability

All data generated or analysed during this study are included in this published article.

## Abbreviations

ADS: Automated driving system
C: Continuity
CAT I: Category I precision approach and landing
CR: Continuity risk
CTI: Continuity time interval
EGNOS: European geostationary navigation overlay service
ERTMS: European railway traffic management system
GBAS: Ground base augmentation system
GNSS: Global navigation satellite system
ICAO: International civil aviation organization
ILS: Instrument landing system
IMO: International maritime organization
IMU: Inertial measurement unit
LPV: Localizer performance with vertical guidance
MTBF: Mean time between failures
MTTF: Mean time to failure
MTTR: Mean time to repair (restore)
PVT: Position, velocity and time
RAMS: Reliability, availability, maintainability and safety
RNP: Required navigation performance
SBAS: Satellite based augmentation system
SIS: Signal-in-space
SoL: Safety-of-life
TLS: Target level of safety
